# Occupational Exposure to *Streptococcus suis* among US Swine Workers

**DOI:** 10.3201/eid1412.080162

**Published:** 2008-12

**Authors:** Tara C. Smith, Ana W. Capuano, Brenda Boese, Kendall P. Myers, Gregory C. Gray

**Affiliations:** University of Iowa College of Public Health, Iowa City, Iowa, USA

**Keywords:** Streptococcus suis, zoonotic disease, swine, occupational health, dispatch

## Abstract

Despite numerous cases of human infection with *Streptococcus suis* worldwide, human disease is rarely diagnosed in North America. We studied 73 swine-exposed and 67 non–swine-exposed US adults for antibodies to *S. suis* serotype 2. Serologic data suggest that human infection with *S. suis* occurs more frequently than currently documented.

*Streptococcus suis* is one of the most important pathogens affecting the swine industry. The gram-positive, encapsulated bacterium causes a wide range of clinical disease syndromes in pigs and other domestic animals. Despite the recognition that *S. suis* infection may result in a life-threatening meningitis or toxic-shock syndrome, little is known about human pathogenesis. A recent outbreak in People’s Republic of China caused by a serotype 2 strain resulted in 38 deaths among 215 infected humans, an 18% mortality rate (*1*). The bacterium has caused sporadic human illness in other countries as well, including the United Kingdom (*2*), and has been identified as a leading cause of bacterial meningitis in Hong Kong Special Administrative Region (*3*) and Vietnam (*4*).

Although human infection with *S. suis* has been occasionally documented in North America (*5*), the first human case was not reported in the United States until 2006, when a farmer from New York sought treatment for meningitis. The source was an area farm where the patient had recently purchased piglets (*6*). Several investigators have suggested that the infrequent diagnosis of human *S. suis* infection is due to underdiagnosis or misdiagnosis, rather than a true absence of disease (*5*,*7*,*8*).

## The Study

To test the hypothesis that human infections with *S. suis* occur more commonly than currently recognized, we examined archived serum samples from 73 swine-exposed and 67 non–swine-exposed adults living in Iowa (*9*). These persons had all previously completed occupational history questionnaires detailing their pig exposure and use of personal protective gear. Use of materials was approved by the Institutional Review Board at the University of Iowa. Antibodies to serotype 2 *S. suis* were measured by an ELISA that used whole *S. suis* cells (serotype 2, strain 30–336–06) as antigen (*10*).

The ELISA optical density readings for the 73 swine-exposed study participants and 67 non–swine-exposed participants were first compared to the positive-control mean option density read per plate per dilution. Optical densities greater or equal to mean positive-control optical density were considered positive. To be conservative, the titer was defined as the last positive dilution before the first negative. Again, when a conservative approach was used, the lowest titer among duplicates was considered as the final antibody titer. We used the Fisher exact test to test the null hypothesis that the exposed group does not have higher incidence of antibody titer >10 than the non–swine-exposed group does. We tested a similar hypothesis for specific risk groups exposed to swine (such as nursing or finishing swine, use of gloves) compared with groups not exposed to swine. Risk factor analyses were performed with exact logistic regression. Seven (9.6%) of 73 swine-exposed study participants were positive, and 1 (1.5%) of the 67 non–swine-exposed participants was positive.

Study participants who work with both finishing and nursery swine had 8.8× the odds of having a titer >10 when compared to nonexposed study participants (exact 95% confidence interval 1.1–406.3). We identified no positive persons in the group that worked solely with nursery swine, a somewhat unexpected finding because most *S. suis* disease occurs in young pigs. However, our study had relatively few persons who worked exclusively with nursery swine (11/73); most participants worked with both nursery and finishing swine. Additionally, no farm-level data on prevalence of *S. suis* where these persons were employed were collected; therefore, whether those persons worked on farms where *S. suis* had been confirmed is not known. Other factors such as age, gender, use of tobacco products, and use of gloves when working with animals were not statistically significant (Table).

**Table:** Characteristics of total swine-exposed study population compared with those who had antibody titers >10 against *Streptococcus suis* serotype 2

Variable	Swine exposed	
	Total (n = 73)	Titer >10 (n = 7)*
Gender		
Male	56	6
Female	17	1
Nursery/finishing swine		
Nursery	11	0
Finishing	1	0
Nursery and finishing	59	7
No	2	0
Years working with swine		
5–10	1	0
>10	70	7
Years living on a swine farm		
0	2	0
5–10	1	0
>10	66	7

*Final antibody titer reflects the lowest positive antibody titer among duplicates. An antibody titer was considered positive when its optical density was greater or equal to mean positive control optical density.

## Discussion

In this cross-sectional pilot study, we found that more swine-exposed persons had higher titers of antibodies to *S. suis* than did non–swine-exposed persons. These data suggest that human infection with *S. suis* is more common in the United States than currently thought.

Two possible reasons stand out regarding the lack of human *S. suis* disease in the United States. One possibility is underdiagnosis or misdiagnosis, rather than a true absence of the disease (*11*). Supporting this hypothesis are reports showing that *S. suis* has been mistaken for enterococci, *Listeria* spp., viridans streptococcus, or *Streptococcus pneumoniae* (*7*,*8*,*11*). Because of this potential misclassification, previous publications have asserted that *S. suis* should be considered in the differential diagnosis of septicemia, especially when complicated by meningitis in adults with a recent history of contact with pigs or unprocessed pork (*12*).

A second possibility is that *S. suis* strains colonizing swine in the United States may be less virulent than Asian strains and therefore unlikely to cause overt human disease even when transferred between species. This possibility is supported by molecular analyses showing that many US strains belong to sequence type (ST) 25, whereas most virulent serotype 2 isolates have been ST1 (*13*). Finally, these hypotheses are not mutually exclusive; both underdiagnosis/misdiagnosis and the circulation of lower-virulence strains may be occurring, resulting in fewer diagnoses of human *S. suis* infections in North America (*11*).

*S. suis* infection is an important occupational disease in humans in many countries. In research conducted in *S. suis*–endemic countries, the annual incidence of *S. suis* meningitis was ≈3 cases/100,000 swine-exposed people: roughly 1,500× higher than the rate in the nonexposed population (*14*). Because of this risk, it has been recommended that persons in daily contact with pigs or pig meat should use protective gloves to avoid skin trauma and subsequent risk for exposure to the bacterium. Because no human vaccine against *S. suis* exists, suitable preventive measures coupled with education and supervision of those who come in contact with live swine or unprocessed pork are important to decrease the transmission of this organism to humans. However, few studies have been conducted to detect subclinical cases of *S. suis* infection; therefore, the true incidence of infection among the swine-exposed is unknown.

Because our findings only examined 1 serotype of *S. suis*, our results may not accurately reflect antibody prevalence. Because we used a whole-cell ELISA, some antibody reactions may be due to cross-reacting antibodies to other serotypes of *S. suis* or other species of *Streptococcus.* However, using a slightly different method and population, other investigators found a higher seroprevalence, particularly among farmers and meat inspectors (*15*). This difference may stem partly from the fact that we used a conservative criterion for considering a sample positive, which may further underestimate seroprevalence in our group. For example, 4 study participants (3 swine exposed) were classified as having a titer <10 because the first dilution (1:10) was negative in 1 repeat test. However, these participants’ serum samples were repeatedly positive in the other 7 dilutions. Acquisition of human positive control serum as a standard to test our assay would enable us to make more definitive comparisons.

Finally, because the samples analyzed for this pilot study were not collected to specifically assess *S. suis* infections, more definitive future prospective studies seem indicated. One limitation of this serologic study is that it does not enable us to distinguish antibodies generated as a result of true infection versus exposure to *S. suis* antigens present in manure or dust in the facility, for example. Additionally, because the questionnaire did not include information on pork consumption or handling of raw pork, those factors could not be examined as potential risks. Future studies might include targeted questionnaires, attempts of bacterial isolation, and serial sera collections to examine serologic evidence of infection.
